# The Law Relating to Medical Officers of Health.—II

**Published:** 1903-06-20

**Authors:** J. E. R. Stephens

**Affiliations:** Barrister-at-Law


### HOSPITAL ADMINISTRATION.

### CONSTRUCTION AND ECONOMICS.

OK

### THE LAW RELATING TO MEDICAL OFFICERS OF HEALTH.—I.

By J. E. R. STEPHENS, Barrister-at-Law.

(*Continued from Page* 141.)

*Offensive Trade.*—Section 21 of the Public Health (London) Act, 1891, enacts that where any manufactory, building, or premises used for any trade, business, process, or manufacture, causing effluvia, is certified to the sanitary authority by their medical officer of health, or by any two legally qualified medical practitioners, or by any ten inhabitants of the district of such authority to be a nuisance or injurious or dangerous to the health of any of the inhabitants of the district, such authority shall make a complaint; and if it appears to the petty sessional court hearing the complaint that the trade, business, process, or manufacture carried on by the person complained of is a nuisance, or causes any effluvia which is a nuisance or injurious or dangerous to the health of any of the inhabitants of the district, then, unless it is shown that such person has used the best practicable means for abating the nuisance, or preventing or counteracting the effluvia, the person so offending (being the owner or occupier of the premises, or being a foreman or other person employed by such owner or occupier) shall be liable to a fine not exceeding £50.

This section applies to any manufacture in which effluvia are given off, though the business may not be an offensive trade within section 19. If the effluvia amount to a nuisance in the sense of causing an annoyance and discomfort, it is sufficient to bring the trade within this section, without proving also that there is injury to health. If the sanitary authority refuse or fail to make the complaint referred to in the above section, the County Council may do so under section 100, or proceedings may be taken against the sanitary authority under section 101.

*Inspection of Dairies.*—It is provided by section 71 of the Public Health (London) Act, 1891, that if the medical officer of health of any district has evidence that any person in the district is suffering from a dangerous infectious disease attributable to milk supplied within the district from any dairy situate within or without the district, or that the consumption of milk from such dairy is likely to cause any such infectious disease to any person residing in the district, such medical officer shall, if authorised by an order of a justice having jurisdiction in the place where the dairy is situate, have power to inspect the dairy, and if accompanied by a veterinary inspector or some other properly qualified veterinary surgeon to inspect the animals therein, and if on such inspection the medical officer of health is of opinion that any such infectious disease is caused from consumption of the milk supplied therefrom, he shall report thereon to the sanitary authority, and his report shall be accompanied by any report furnished to him by the said veterinary inspector or veterinary surgeon, and the sanitary authority may thereupon serve on the dairyman notice to appear before them within such time, not less than 24 hours, as may be specified in the notice, to show cause why an order should not be made requiring him not to supply any milk therefrom within the district until the order has been withdrawn by the sanitary authority. Section 141 defines the expression "dairy" as including any farm, farmhouse, cowshed, milk store, milk shop, or other place from which milk is supplied, or in which milk is kept for purposes of sale. The dairy may be in another county, and in that case the order must be made by a justice of that county.

*Notification of Infectious Diseases.*—Section 55 of the Public Health (London) Act, 1891, provides that where an inmate of any house within the district of a sanitary authority is suffering from an infectious disease to which this section applies, notice thereof shall be sent to the medical officer of health of the district. Every medical practitioner attending on or called in to visit the patient shall forthwith on becoming aware that the patient is suffering from an infectious disease to which this section applies send to the medical officer of health of the district a certificate stating the full name and the age and sex of the patient, the full postal address of the house, and the infectious disease from which in the opinion of such medical practitioner the patient is suffering, and stating also whether the case occurs in the private practice of such practitioner or in his practice as a medical officer of any public body or institution; and where the certificate refers to the inmate of a hospital it shall specify the place from which and the date at which the inmate was brought to the hospital, and shall be sent to the medical officer of health of the district in which the said place is situate. Every person required by this section to send a notice or certificate who fails forthwith to send the same shall be liable to a fine not exceeding £2. The sanitary authority shall gratuitously supply forms of certificate to any medical practitioner residing or practising in their district who applies for the same, and shall pay to every medical practitioner for each certificate duly sent by him in accordance with this section a fee of 2s. 6d. if the case occurs in his private practice, and of 1s. if the case occurs in his practice as medical officer of any public body or institution. Where a medical officer of health receives a certificate under this section relating to a patient within the Metropolitan Asylum District, he shall within twelve hours after such receipt send a copy thereof to the Metropolitan Asylum managers, and to the head teacher of the school attended by the patient (if a child), or by any child who is an inmate of the same house as the patient. The Metropolitan Asylum managers shall repay to the sanitary authority the fees paid by that authority in respect of the certificates whereof copies have been so sent to the managers. The managers shall send weekly to the County Council and to every medical officer of health such return of the infectious diseases of which they receive certificates in pursuance of this section as the County Council require. The duty of forwarding these copies devolves on the medical officer of health, and it is apparently his duty to make inquiry in every case whether there is in the house where the patient is any child attending school, and at what school such child attends.

Where in any district of a sanitary authority there are two or more medical officers of health of that authority, a certificate under this section shall be sent to such one of those officers as has charge of the area in which is the patient referred to in the certificate, or to such other of hose officers as the sanitary authority may direct. This subsection applies only to the certificate of the medical practitioner. A notice or certificate to be sent to a medical officer in pursuance of this section may be sent to such officer at his office or residence.

The above section applies to every building, vessel, tent, van, shed, or similar structure used for human habitation in like manner as nearly as may be as if it were a house. In this section the expression "infectious disease to which this section applies" means any of the following diseases, namely, small-pox, cholera, diphtheria, membranous croup, erysipelas, the disease known as scarlatina or scarlet fever, and the fevers known by any of the following names: typhus, typhoid, enteric, relapsing, continued, or puerperal, and includes as respects any particular district any infectious disease to which this section has been applied by the sanitary authority of the district.

Section 73 of the Public Health (London) Act, 1891, provides that if a person dies in a hospital from any dangerous infectious disease, and the medical officer of health, or any legally qualified medical practitioner, certifies that, in his opinion, it is desirable, in order to prevent the risk of communicating such infectious disease, that the body be not removed from such hospital except for the purpose of being forthwith buried, it shall not be lawful for any person to remove the body except for that purpose; and the body when taken out of such hospital shall be forthwith taken direct to the place of burial and there buried. If any person wilfully offends against this section he shall, on the information of the sanitary authority, be liable to a fine not exceeding £10.

*Unsound Food.*—Section 47 of the Public Health (London) Act, 1891, provides that any medical officer of health or sanitary inspector may at all reasonable times enter any premises and inspect and examine (*a*) any animal intended for the food of man which is exposed for sale, or deposited in any place for the purpose of sale, or of preparation for sale; and (*b*) any article, whether solid or liquid, intended for the food of man, and sold or exposed for sale or deposited in any place for the purpose of sale or of preparation for sale. The proof that the same was not exposed or deposited for any such purpose or was not intended for the food of man, resting with the person charged; and if any such animal or article appears to such medical officer or inspector to be diseased, or unsound, or unwholesome, or unfit for the food of man, he may seize and carry away the same himself or by an assistant in order to have the same dealt with by a justice.

The question whether any given time is reasonable or not for entering premises to inspect must depend upon the circumstances of each case. In *Small* v. *Bickley* (39 J.P. 442) the appellant, a butcher, while at his residence, half a mile from his shop, on a Sunday afternoon was requested to go himself or send someone with the key to admit the inspector of nuisances to his shop in order that some meat there might be examined. He refused, and was convicted under 26 & 27 Vict., c. 117, section 3, of preventing, obstructing, or impeding the inspector when duly engaged in carrying into execution the provisions of the Act. It was held that although Sunday afternoon might, under some circumstances, be a reasonable time for examining meat, the appellant, at most, had only refused to assist the inspector, and had not prevented, obstructed, or impeded him, and that the conviction was wrong. The word "place" in the above section is used in a general sense. Two carcases of cows unfit for food were found in a yard at the back of a butcher's house, there being a slaughter-house on one side of the yard. It was held that the yard was a "place" within the corresponding words in 26 and 27 Vict. c. 117, section 2 (*Young* v. *Gratridge* 33 J.P. 260). Under the same Act it was held that diseased meat placed upon a cart, when passing along the streets of Dublin from a slaughter-house to a place for the manufacture of preserved meats was properly seized (*Daly* v. *Webb* 4 Ir. R.C.L. 309). According to the opinion of Drs. Letheby and Tidy, given in evidence in a case reported in 37 J.P. 267, an animal killed immediately before, or during, or after parturition is unfit for human food. It was held under the corresponding sections of the Public Health Act, 1875, that meat might be taken before a justice and condemned without any summons or notice to the person to whom it belonged, and that such person having been, subsequently to the destruction of the meat, summoned and convicted, such conviction was good (*White* v. *Redfern*, 44 J. P. 87). In *R.* v. *Blount* (43 J. P. 383) the defendant was charged with having unlawfully exposed or deposited for sale or preparation for sale certain meat intended for human food. The magistrate dismissed the charge on the ground that the defendant was himself unaware of the fact that the meat was upon his premises, as it had arrived there and been seized in his absence. Subsequently, a summons was issued against the defendant in respect of the same seizure for having on his premises meat for the purpose of sale, etc., and the same evidence was given as that adduced on the first charge. The defendant was convicted on the second summons, but the court quashed the conviction on the ground that the defendant might have been convicted of the offence charged on the second summons when he appeared on the first, and that the second summons was for the same matters. It will be advisable, therefore, in issuing a summons to confine it to one offence, otherwise a conviction founded upon it may be had for duplicity. In *Vinter* v. *Hind* (47 L. P. 373) the respondent, a butcher, exposed for sale part of a cow which had died of disease, and sold the meat to a customer, who took it home for food, and some days afterwards at the request of the appellant, an inspector of nuisances, handed it over to him, and it was condemned by a justice as unfit for the food of man. It was held that the meat was not so seized and condemned as is prescribed by section 116, and that the defendant could not for that reason be convicted under section 117 of the Public Health Act, 1875. In *Barlow* v. *Terrett* (L. R. 1891, 2 Q. B. 107) the appellant, a farmer in the country, sent to a salesman in London meat which, to his knowledge, was unsound for the purpose of its being sold and used as human food. The salesman did not expose the meat for sale, but put it aside, and called the attention of the respondent, an inspector of nuisances, to it. The respondent seized the meat, and obtained a justice's order for its destruction. The appellant having been convicted of being the owner of unsound meat, unlawfully deposited for the purpose of sale and intended for the food of man, it was held that, in order to convict the owner of an offence under the corresponding provision in the Nuisances Removal Act, there must be either a sale or an exposure of meat for sale, and that the conviction was therefore bad.

If any person obstructs an officer in the performance of his duty under any warrant for entry into any premises granted by a justice in pursuance of this Act, for the purposes of this section, he shall, if the court is satisfied that he obstructed with intent to prevent the discovery of an offence against this section or has within 12 months previously been convicted of such obstruction, be liable to imprisonment for any term not exceeding one month in lieu of any fine authorised by this Act for such obstruction. Where a person has in his possession any article which is unsound or unwholesome, or unfit for the food of man, he may, by written notice to the sanitary authority, specifying such article, and containing a sufficient identification of it, request its removal, and the sanitary authority shall cause it to be removed as if it were trade refuse.

